# Reflections and aspirations: the journal after 5 years

**DOI:** 10.1186/s13643-018-0753-2

**Published:** 2018-06-20

**Authors:** David Moher, Lesley A. Stewart, Paul Shekelle

**Affiliations:** 1 Clinical Epidemiology Program, Ottawa Hospital Research Institute, General Campus, 501 Smyth Road, Room L1288, Ottawa, ON K1H 8L6 Canada; 2 Centre for Reviews and Dissemination, University of York, Heslington, York, UK; 3 West Los Angeles VA Medical Center, Los Angeles, CA USA

## Abstract

The journal recently celebrated its fifth anniversary. Like systematic reviews themselves, the journal is thriving and publishing a variety of protocols, reviews, and methods papers. We have also had success in publishing-themed series.

Systematic Reviews in now 5 years old—we launched in 2012 at a time of rapid growth in the number of systematic reviews. In 2009, it was estimated that 11 new systematic reviews were published daily [1]. By 2016, the estimate more than doubled to 28 systematic reviews daily [2]. The first paper published by the journal reported on the development of PROSPERO, an international prospective register of systematic reviews [3]. Since then, we have been humbled by the interest the systematic review community and others have taken in the journal. In 2017, almost three quarters of a million people accessed the journal. Our reach is global with corresponding authors from over 50 countries. As of the end of 2017, we had published 983 articles.

Publications are important for many reasons. They share knowledge with many groups including patients and the public, particularly so for open access articles where there are no financial barriers preventing anyone from reading research findings. For many authors, metrics associated with publishing journal articles continue to be an important component used in decisions about how they are hired, promoted, and tenured. We are delighted that our publisher has signed the Declaration of Research Assessment (DORA). One of the strongest messages DORA makes is for assessment committees not to use journal impact factors in their evaluations of scientists [4]. Other metrics, such as author citations, can be used as part of the toolbox for assessing researchers. The journal’s publications have been cited over 3200 times; our 2017 journal citation distribution [5] can be seen in Fig. 1. The distribution indicates that most articles published during 2015 and 2016 have been cited a few times. For example, 50 articles were cited twice. The “more” column at the end of the long tail of the figure is for one of the journal’s articles cited 617 times. Assessors can also use open science practices to gauge researchers, such as registration of study protocols, including systematic reviews, sharing of data, materials, and methods.

**Fig. 1:**
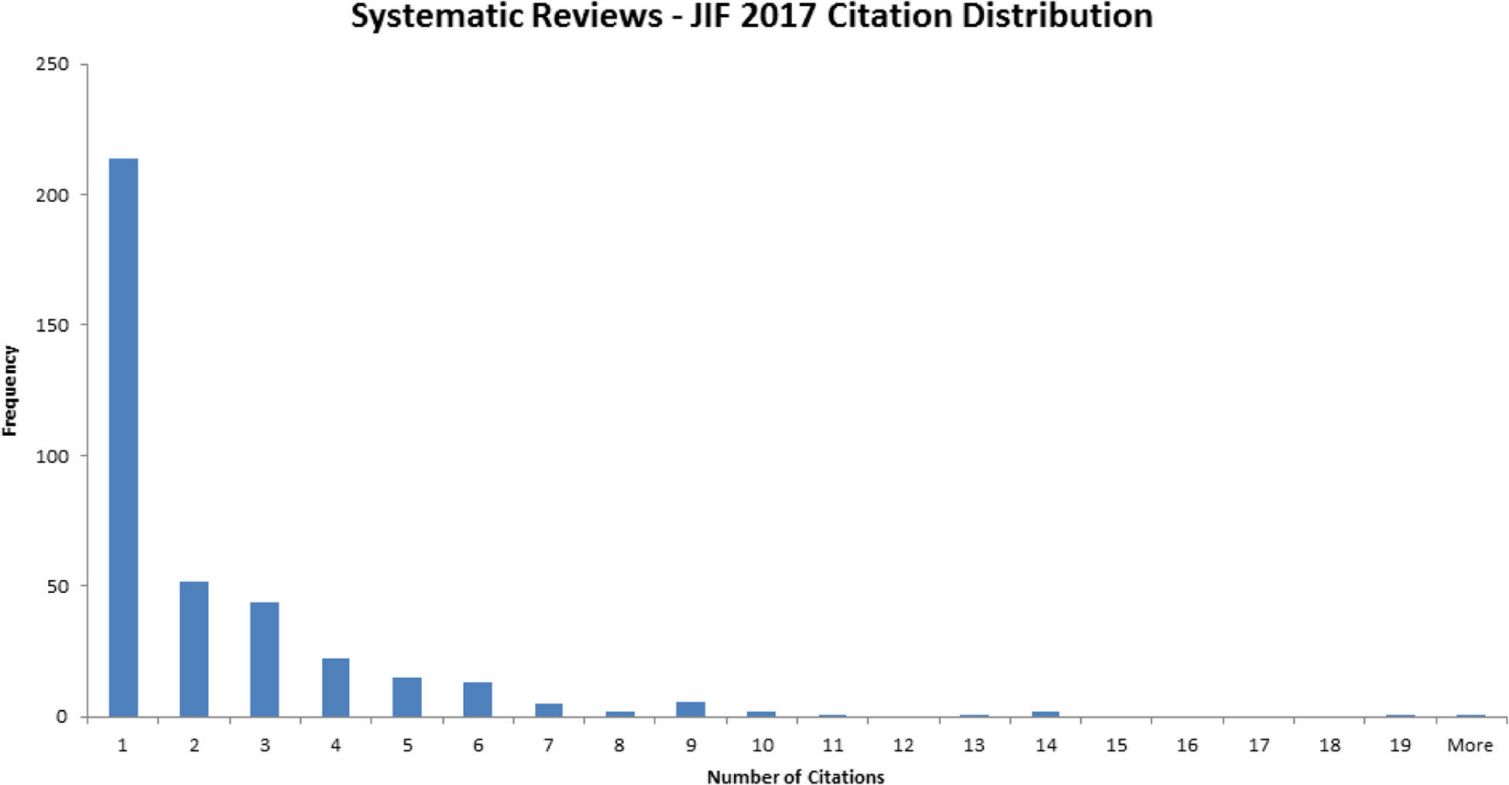
Systematic Reviews - 2017 journal citation distribution

The world of systematic reviews seemed a little simpler 5 years ago. Few modifications of traditional pairwise systematic reviews with pairwise meta-analysis were published. Today, there is a burgeoning array of review types some of which the journal publishes: rapid reviews, realist reviews, network meta-analysis, scoping reviews, and overviews. With these developments, we have often reflected on whether the field is clear about what constitutes a systematic review. In our opinion, some of the newer review types appear to abut or cross into how we have traditionally defined a systematic review as “a review of a clearly formulated question that uses systematic and explicit methods to identify, select, and critically appraise relevant research, and to collect and analyze data from the studies that are included in the review” [6]. When the journal receives manuscripts adhering to these criteria, it is easy for the editors to categorize them as systematic reviews. Sometimes, the journal receives manuscripts, labeled as systematic reviews, which meet some of these criteria, such as critical appraisal and well-developed searches, but not others. The latter often appear to be closer to scoping reviews defined as “aim to map rapidly the key concepts underpinning a research area and the main sources and types of evidence available, and can be undertaken as stand-alone projects in their own right, especially where an area is complex or has not been reviewed comprehensively before” [7]. When submitting scoping reviews to the journal, we encourage authors to declare them as such and not describe them as systematic reviews.

In its first 5 years, the journal also published eight themed series (https://systematicreviewsjournal.biomedcentral.com/articles/collections). The first focused on the importance of registering systematic reviews. The current series is devoted to overviews of systematic reviews, and we have recently issued a call for papers for a series on automation in systematic reviews. All of journal’s efforts are aimed at keeping to what the journal is consistently trying to achieve, namely, advancing the discourse regarding systematic reviews. We want all of our publications reported in a clear and transparent way such that interested readers can replicate both methods and results.

For 2016, the latest available data, the time from submission to initial decision was 46 days and time from acceptance to publication was 14 days. While we strive to ensure the journal operates smoothly and efficiently, we recognize there are failures and we sometimes keep authors waiting for too long. We continue to work hard to improve these times and we welcome innovative ideas from readers. The journal’s associate editors are an integral part of the journal’s successes. We started the journal with small group of dedicated associate editors; today, we have over 30 of them. Our associate editors are one of the cornerstones of the journal and part of our success. Similarly, there are thousands of peer reviewers who have provided us with insight and help in our decision-making. Full annual lists of our reviewers can be found via the Reviewer Acknowledgement page on our website: https://systematicreviewsjournal.biomedcentral.com/reviewer-acknowledgements. Without this invisible college, the journal (and most others) would be lost.

It is easier looking back compared to reading tealeaves regarding the journal’s next 5 years. First and foremost, we will continue to work hard making the journal more efficient. We anticipate timelier processing of submissions and decision-making about their outcome while maintaining the highest possible standards. Although there have been improvements in the reporting of systematic reviews, there is still room for improvement and work to be done to ensure they are completely, transparently, and clearly reported [2]. Whether authors are submitting methods articles, protocols of reviews, or any of the various types of systematic reviews, we recommend use of reporting guidelines, all of which can be found on the EQUATOR Network’s library.

Exciting developments are afoot, the systematic review community is likely on the cusp of harnessing technology to automate parts of the systematic review process. Over the past few years, network meta-analyses have brought real innovation and helped us to address real-world health care questions of “what works best” that pairwise reviews could not easily address. We continue to welcome submissions of network meta-analysis protocols and completed ones. Living systematic reviews have entered the stage and their methods and reporting will likely continue to be refined. These reviews pose interesting methodological challenges and publishing ones as well, and we are keen to publish on their methodological developments and finding innovative ways to publish living reviews.

The broader research community has spoken about the importance of data sharing (i.e., data, code, and materials). The individual patient data meta-analysis community has been leaders here [8]. The journal and publisher is also committed to data sharing. We support the Transparency and Openness Promotion guidelines [9]. We encourage authors to submit any underlying data when submitting articles with data. Such sharing can also promote reproducibility of methods and results. The journal, and publisher, has implemented a Research Data Support Services to help authors in this regard; although, we also welcome sharing data in other ways such as through the Systematic Review Data Repository [10].

Three years ago, we expressed our views on the increasing family of reviews [11]. The journal still maintains its openness to publishing variants of systematic reviews, such as rapid reviews. Methods about the conduct and reporting of reviews continue to develop. We will continue to monitor these. In the meantime, we encourage authors to think of the journal when considering a home for the increasing range of types of systematic reviews. The broader research community has spoken about the importance of data sharing. We hope to see more data sharing from authors publishing in the journal.

The past 5 years have seen many developments and extensions of systematic review methods and expansion in existing into new areas of research. We hope that *Systematic Reviews* has played a role in supporting evidence synthesis and the evidence synthesis community and that the journal will continue to enrich its readership in the coming years.
